# Single-cell genomic profiling of antimicrobial resistance in *Escherichia coli* from the Densu River, Ghana

**DOI:** 10.3389/fmicb.2026.1797725

**Published:** 2026-04-23

**Authors:** Runa Furuya, Yohei Nishikawa, Yusuke Ota, Isaac Prah, Samiratu Mahazu, Masako Kifushi, Mitsunori Yoshida, Masato Suzuki, Yoshihiko Hoshino, Toshihiko Suzuki, Haruko Takeyama, Anthony Ablordey, Ryoichi Saito

**Affiliations:** 1 Department of Molecular Microbiology and Immunology, Graduate School of Medicine and Dental Science, Institute of Science, Tokyo, Japan; 2 Biomanufacturing Process Research Center, National Institute of Advanced Industrial Science and Technology (AIST), Ibaraki, Japan; 3 Research Organization for Nano & Life Innovation, Waseda University, Tokyo, Japan; 4 Department of Life Science and Medical Bioscience, Graduate School of Advanced Science and Engineering, Waseda University, Tokyo, Japan; 5 Department of Mycobacteriology, Leprosy Research Center, National Institute of Infectious Diseases, Tokyo, Japan; 6 Antimicrobial Resistance Research Center, National Institute of Infectious Diseases, Tokyo, Japan; 7 Department of Bacterial Pathogenesis, Infection and Host Response, Institute of Science, Tokyo, Japan; 8 Institute for Advanced Research of Biosystem Dynamics, Waseda Research Institute for Science and Engineering, Graduate School of Advanced Science and Engineering, Waseda University, Tokyo, Japan; 9 Department of Bacteriology, Noguchi Memorial Institute for Medical Research, Accra, Ghana

**Keywords:** antimicrobial resistance, aquatic environment, phylogenetic analysis, single-cell genomic analysis, virulence factors

## Abstract

**Introduction:**

River water serves as a natural reservoir for antimicrobial resistance (AMR) factors. Although environmental AMR poses a global threat to public health as it spreads to local communities through the microbiome in aquatic environments, the actual situation remains unclear, especially in developing countries. In this study, we sought microbiome data, including AMR information, for multiple bacterial strains from river water samples using a single-cell genomics platform.

**Methods and results:**

After antimicrobial selection of samples from the Densu River in Ghana, 16S rRNA amplicon sequencing revealed a high proportion of the genus *Escherichia-Shigella* with ampicillin and sulbactam selection. Single-cell genomic analysis revealed differences in AMR and virulence factor profiles among the same species of *Escherichia coli*, including the CTX-M-15 extended-spectrum *β*-lactamase-producing ones. Pan-genome analysis predicted 4,814 gene clusters, of which 2,264 were accessory, including 605 singletons. Phylogenetic tree analysis using the maximum likelihood method showed the heterogeneity of single-cell amplified genomes (SAGs), and cluster of orthologous gene analysis for each SAG confirmed the difference in the ratio of each functional group.

**Conclusion:**

This study demonstrates the potential of single-cell genomics using the single-cell amplified genome in gel method to enhance environmental AMR surveillance with high resolution and accuracy. It also represents the first application of this approach to aquatic environments in Ghana, thereby contributing to the development of microbial ecology and genomic resources.

## 1 Introduction

Antimicrobials are widely used to treat bacterial infections; however, bacteria continuously evolve mechanisms to resist and survive under selective pressure. The increasing and often unregulated use of antimicrobials, particularly in developing countries, has accelerated the emergence and dissemination of antimicrobial resistance (AMR), leading to significantly higher morbidity and mortality rates. In recent years, water environments, including hospital and municipal wastewater, natural waterways, sediments, and biofilms, have served as significant reservoirs for AMR, with studies highlighting widespread global contamination and potential for human exposure through recreational activities, drinking water, and environmental contact, warranting the urgent need for longitudinal monitoring and a One Health approach to mitigate public health risks (Mills and Lee, 2019). In Sub-Saharan Africa, including Ghana, the mortality rate has been the highest at 27.3 deaths per 100,000 people (May, 2023). Moreover, the presence of shared antimicrobial resistance genes (ARGs) across human, animal, and environmental sources in Ghana (Donkor et al., 2025) highlights the need for a One Health approach. The Densu River Basin in southern Ghana, with a population density of approximately 460 persons per square kilometer, is facing increasing ecological stress due to urbanization and agricultural expansion. A limited wastewater treatment infrastructure has led to the frequent discharge of untreated effluents into the river, contributing to water quality degradation and creating conditions conducive to the spread of AMR determinants (Ofosu et al., 2020). In a previous study, we detected carbapenem-resistant bacteria from a river in Ghana using the culture-based method (Ofosu Appiah et al., 2025), demonstrating the significance of comprehensive AMR surveillance, especially for carbapenemase-producing and extended-spectrum *β*-lactamase (ESBL)-producing bacteria in river water.

AMR evaluation has traditionally relied on culture-based methods, which allow the isolation and phenotypic characterization of individual bacterial strains. Although standardized, these methods are limited by their inability to detect non-culturable microorganisms. Recent studies have shown that even major pathogens such as *Escherichia coli* can enter a viable but non-culturable state under environmental stressors, such as ultraviolet (UV) exposure or chlorination (Zhang et al., 2024). This phenomenon challenges the reliability of culture-based surveillance, especially in environmental contexts where most microorganisms are non-culturable (Lloyd et al., 2018). To overcome these limitations, metagenomic approaches are essential for profiling microbial communities and their associated AMR traits. Although metagenomic analysis enables the characterization of genomic composition at the population and community levels, it remains challenging to resolve evolutionary dynamics at the subpopulation or strain level or to determine their genomic context (i.e., chromosomal vs. plasmid localization; Bawn et al., 2022). To address these obstacles, single-cell genomics has emerged as a powerful tool that enables the simultaneous acquisition of microbial taxonomy, ARGs, and virulence factors from individual cells. This approach allows for the direct association of functional traits with specific microbial genomes, thereby offering novel insights into the ecology and health risks of environmental microbiomes. Despite its promise, the application of single-cell genomics remains limited, owing to insufficient environmental datasets, particularly in aquatic ecosystems with low biomass.

In the single-cell amplified genome in gel (SAG-gel) method, individual microbial cells are selectively or randomly encapsulated into picoliter-volume droplets from a population, cooled to generate gel beads, collected in tubes, lysed, and their genomes are amplified using whole-genome amplification (WGA) techniques (Chijiiwa et al., 2020). This approach offers several advantages, such as the ability to identify heterogeneity within a bacterial community, including closely related species, and high-quality data that are not affected by sample diversity. To the best of our knowledge, there have been no reports on the use of single-cell genomics for AMR detection in river water samples from Ghana. In this study, we employed a single-cell genomics approach to simultaneously obtain high-resolution data on microbial taxonomy, ARGs, and virulence factors from river water samples collected from the Densu River Basin, Ghana. This method enabled the direct association of AMR and virulence traits with individual microbial genomes, providing novel insights into the ecology and potential health risks of environmental microbiomes.

## 2 Materials and methods

### 2.1 Sample collection and enrichment of AMR bacteria

A 100 mL water sample was collected from the Densu River (5°54′42″N, 0°19′14 W) in Ghana on June 14, 2021, which contained 1.40 × 10^7^ bacterial cells, and was subsequently filtered through 150- and 50-μm nylon meshes, followed by filtration using two 5 μm filters, a 0.8 μm filter, and finally a 0.45 μm Sterivex filter (Merck Millipore Ltd., County Cork, Ireland). A portion of filtrate (~1/4 of the volume) collected through the 0.45 μm membrane filter was transferred into 2 mL of tryptic soy broth supplemented with 32 μg/mL ampicillin and 16 μg/mL sulbactam (A/S), vortexed thoroughly, and incubated at 37 °C overnight to enrich for *β*-lactamase-producing bacteria.

### 2.2 16S rRNA gene amplicon sequencing

DNA was extracted from samples enriched in tryptic soy broth with or without A/S, using a NucleoSpin Tissue kit (Takara Bio, Shiga, Japan). The V3–V4 regions of the 16S rRNA genes were analyzed according to the Illumina protocol for 16S Metagenomic Sequencing Library Preparation (Illumina, San Diego, CA, United States). The initial polymerase chain reaction (PCR) was carried out in a reaction mixture containing the KAPA HiFi HotStart polymerase (Kapa Biosystems, Wilmington, MA, United States) with 341F and 806R primers (5′-TCGTCGGCAGCGTCAGATGTGTATAAGAG ACAGCCTACGGGNGGCWGCAG-3′ and 5′-GTCTCGTGGG CTCGGAGATGTGTATAAGAGAC AGGACTACHVGGGTATCT AATCC-3′), under the following thermal cycling conditions: an initial denaturation at 95 °C for 3 min; 32 cycles of denaturation at 95 °C for 30 s, annealing at 55 °C for 30 s, and extension at 72 °C for 30 s; and a final extension at 72 °C for 5 min. A second PCR was performed using the same polymerase and a different primer set (5′-AATGATACGGCGACCACCGAGATCTACAC-(index sequence)-TCGTCGGCAGCGTC-3′ and 5′-CAAGCAGAAG ACGGCATACGAGAT-(index sequence)-GTCTCGTGGGCTCG G-3′), with the following conditions: initial denaturation at 95 °C for 3 min; 8 cycles of denaturation at 95 °C for 30 s, annealing at 55 °C for 30 s, and extension at 72 °C for 30 s; and a final extension at 72 °C for 5 min. The libraries were sequenced on an Illumina MiSeq instrument using 301 bp × 2 paired-end reads and the MiSeq Reagent Kit v3 with 10% phiX. Raw sequencing reads were processed using the DADA2 pipeline (Callahan et al., 2016) for noise removal and clustering. The taxonomic classification of the resulting amplicon sequence variants was performed using the SILVA reference database (Quast et al., 2012).

### 2.3 Single-cell genome sequencing

Bacterial single-cell genome sequencing was performed using the SAG-gel platform as described previously (Nishikawa et al., 2022). Briefly, bacterial cells from river water samples incubated in tryptic soy broth supplemented with A/S were centrifuged and washed three times with Dulbecco’s phosphate-buffered saline (−; DPBS, Thermo Fisher Scientific). After determining the cell concentration, the samples were mixed with 1.5% ultra-low gelling temperature agarose (A5030, Merck, Darmstadt, Germany) in DPBS and adjusted to a concentration of 0.3 cell/droplet (30 μm diameter). Agarose gel droplets were generated using the On-chip Droplet Generator (On-chip Biotechnologies, Tokyo, Japan) and solidified on ice.

The solidified gel beads were recovered from the oil phase by sequential washing with 1H,1H,2H,2H-perfluoro-1-octanol (Sigma-Aldrich), acetone (Sigma-Aldrich), and isopropanol (Sigma-Aldrich). After resuspension of the gel beads in DPBS, the bacterial cells encapsulated in gel beads were lysed by overnight incubation at 40 °C in proteinase K solution (1 mg/mL proteinase K [Promega, Madison, WI] and 0.5% SDS [Wako, Tokyo, Japan] in DPBS). Whole-genome amplification was performed using a REPLI-g Single Cell Kit (Qiagen, Hilden, Germany) at 30 °C for 3 h. Genome amplification within the gel beads was confirmed using 1 × SYBR Green I (Thermo Fisher Scientific), and fluorescence-positive gel beads were isolated using a BD FACSMelody Cell Sorter (BD Biosciences) equipped with a 488 nm excitation laser.

A total of 242 fluorescence-positive gel beads were sorted into a 384-well microplate, and next-generation sequencing libraries were prepared using the QIAseq FX DNA library kit (QIAGEN). Each single-amplified genome (SAG) library was sequenced on an Illumina NextSeq 2000 platform with 150 bp × 2 paired-end reads (Illumina). The sequence raw reads were quality filtered using BBDuk v38.90 (options: qtrim = r trimq = 10 minlength = 40 maxns = 1 minavgquality = 15 ktrim = r ref. = adapters k = 23 mink = 11 hdist = 1 tpe tbo; Kifushi et al., 2024). The quality-filtered reads were assembled *de novo* using SPAdes v3.9.0 (Bankevich et al., 2012), and assembly statistics were evaluated using QUAST v4.5 (Gurevich et al., 2013). Genome completeness and contamination were assessed using CheckM v1.0.6 (Parks et al., 2015).

### 2.4 Whole-genome sequencing

DNA was extracted from the river water sample enriched in tryptic soy broth with A/S, using the MagAttract HMW DNA Kit (Qiagen). Whole-genome sequencing was performed using the DNBSEQ platform. Genomic DNA quality was first assessed prior to library preparation. Sequencing libraries were prepared from 100 ng of genomic DNA using the MGIEasy FS DNA Library Prep Set (MGI Tech, Shenzhen, China) according to the manufacturer’s protocol. DNA was enzymatically fragmented for 9 min, followed by a two-step size selection using magnetic beads to obtain fragments with an insert size of approximately 300 bp. Fragmented DNA was subjected to end repair and A-tailing, followed by adapter ligation. The adapter-ligated DNA fragments were amplified by PCR for 8 cycles and purified. The purified libraries were heat-denatured and circularized to generate ssDNA (single-stranded DNA). Equal amounts of ssDNA from each sample were pooled and used to prepare DNA nanoballs (DNBs). The DNB libraries were loaded onto a flow cell and sequenced using the DNBSEQ-G400RS platform with the DNBSEQ-G400 High-throughput Sequencing Set and an FCL flow cell (MGI Tech). Paired-end sequencing was performed with a read length of 150 bp × 2.

### 2.5 Sequencing-based analysis

Gene prediction was performed using eggNOG-mapper v2.1.12 (Cantalapiedra et al., 2021), and clusters of orthologous gene (COG) categories or pathways were analyzed using the obtained COG IDs. Pan-genome analysis was performed using Anvi’o v8 (Eren et al., 2020). Taxonomic annotation of draft genomes was performed using GTDB-Tk v1.7.0 (Chaumeil et al., 2020). The ARGs and plasmids were predicted using Staramr v0.7.2 (Bharat et al., 2022). Virulence factors were identified using VirulenceFinder 2.0 (Joensen et al., 2014). The average nucleotide identity (ANI) values of the selected SAGs were calculated using FastANI v1.34 (Jain et al., 2018). A maximum-likelihood phylogenetic tree was constructed using kSNP4.1 (Hall and Nisbet, 2023) and visualized using iTOL v7 (Letunic and Bork, 2024).

## 3 Results

### 3.1 High prevalence of *Escherichia coli* in the river sample under antimicrobial pressure

**Figure 1:**
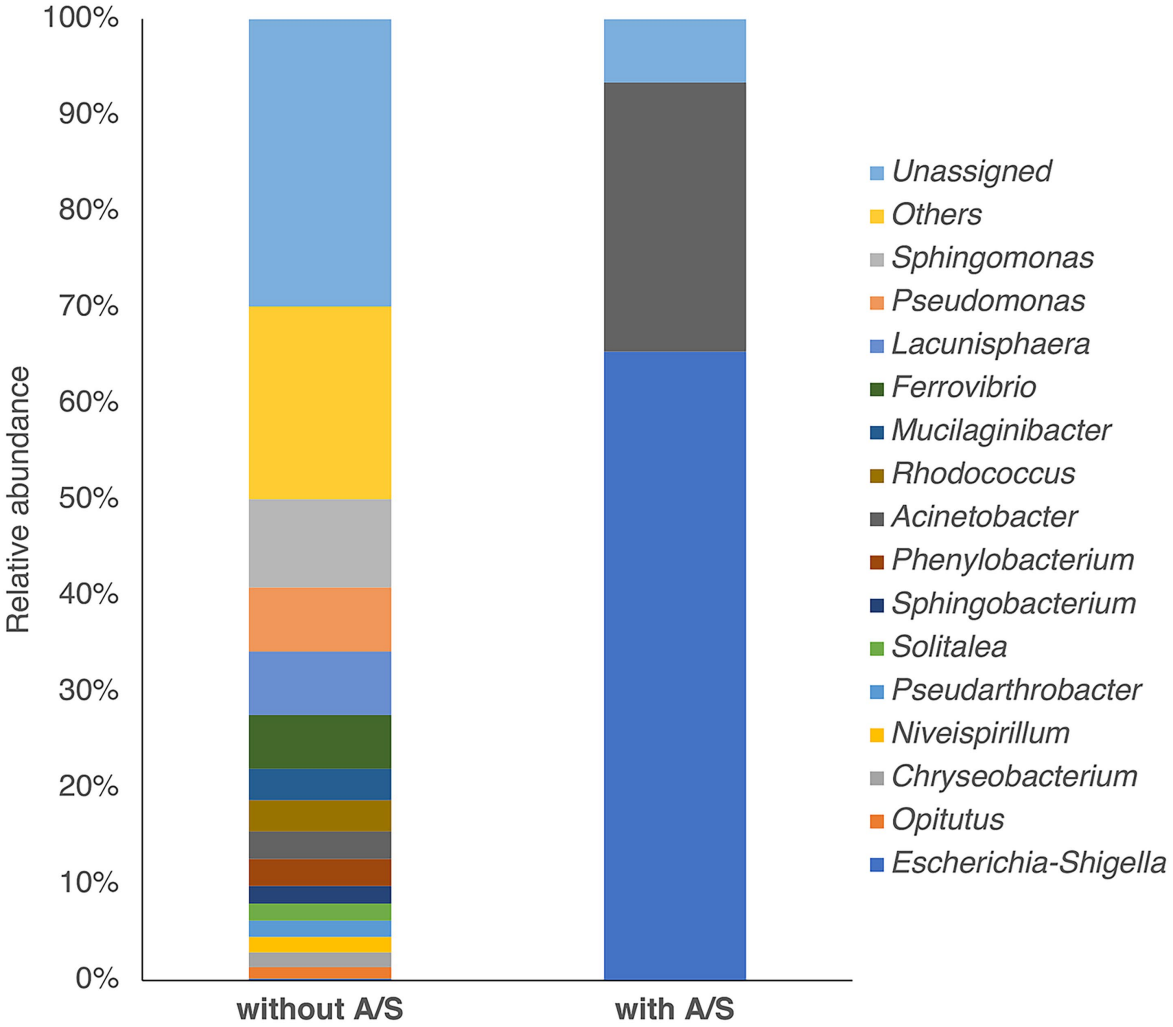
Bacterial composition at the genus level determined by 16S rRNA amplicon sequencing of river samples enriched in tryptic soy broth with or without 32 μg/mL ampicillin and 16 μg/mL sulbactam (A/S).

We conducted 16S rRNA gene amplicon sequencing to analyze the microbiome of the river sample, with or without antimicrobial selection, to assess the diversity of bacterial species harboring ESBL and carbapenemase genes. The genus *Sphingomonas* was predominant in the original sample. The sample selected with A/S showed 65.4% abundance of the genus *Escherichia-Shigella* and 28.8% of the genus *Acinetobacter* (Figure 1). The data summary of 16S rRNA amplicon sequencing is provided in Supplementary Table 1. Next, we conducted single-cell genome sequencing of the samples using A/S and identified the bacterial taxa. Of the 242 SAGs, 158 were classified as medium-quality draft SAGs (Bowers et al., 2017). The average completeness and contamination of the SAGs were 51 and 2%, respectively; the average genome size and N50 were 2.47 Mb and 7.49 kb, and the average number of contigs was 1,979. The detailed QC metrics are provided in Supplementary Table 2. Of the 242 generated SAGs, 185 SAGs had assigned taxonomies at the species level, and 94.6% of these SAGs were classified as *Escherichia flexneri* based on the GTDB-Tk taxonomy. The remaining were classified as *E. coli*, *Escherichia dysenteriae*, and *Pseudomonas otitidis*. The representative *E. flexneri* SAG (GH9_AS_000016), which had the highest completeness of 97.75% among the SAGs, showed the highest ANI score (97.00%) with the *E. flexneri* reference genome (GCF_002950215.1), but in the pairwise ANI analysis, it showed a higher ANI score (99.63%) with the *E. coli* complete genome (CP018995). In the GTDB, approximately 80% of the genomes previously annotated as *E. coli* were reassigned to *E. flexneri* or *E. dysenteriae* (Parks et al., 2021). Notably, the model laboratory strain, *E. coli* K-12, was grouped with *E. flexneri*, reflecting its closer genomic affinity to the *E. flexneri* type strain than to *E. coli*. Therefore, we considered that these SAGs belonged to *E. coli* and have focused on *E. coli*, hereafter.

### 3.2 Phylogenetic and AMR features of *Escherichia coli* genomes

**Figure 2:**
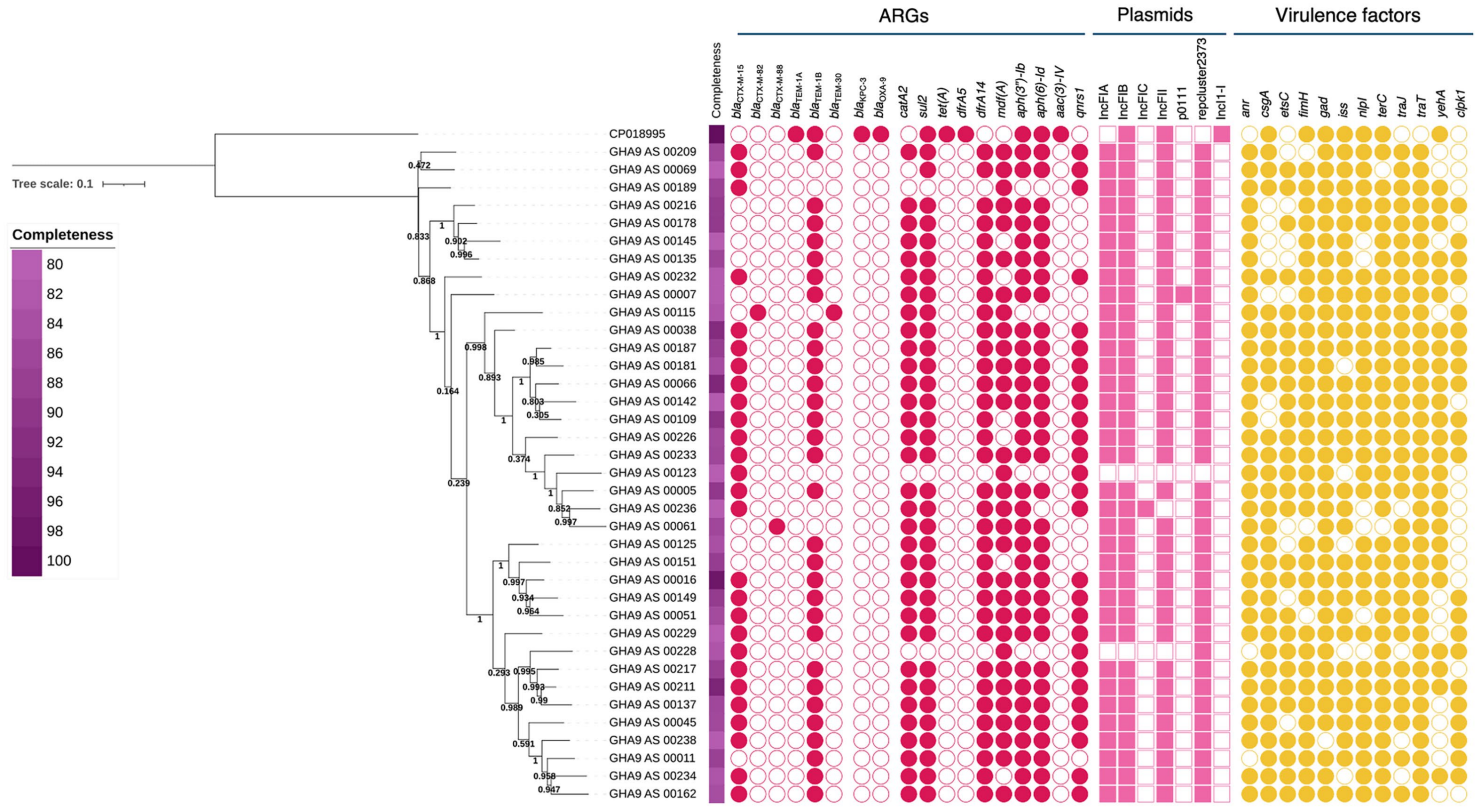
Phylogenetic tree using a single-nucleotide polymorphism-based maximum-likelihood method along with their profiles of antimicrobial resistance (AMR) genes, plasmids, and virulence factors. Thirty-seven single-cell amplified genomes (SAGs) with completeness greater than 80% and contamination less than 5% were used. Bootstrap values inferred from 1,000 replicates are indicated at the nodes, and values ≥95% were considered robust. The presence or absence of specific ARGs, plasmids, and virulence factors is shown on the right. ARGs: Antibiotic resistance genes.

Using 37 SAGs classified as *E. coli* with completeness >80% and contamination <5% corresponding to medium-quality SAGs (Bowers et al., 2017), we performed a phylogenetic analysis using a single-nucleotide polymorphism-based maximum-likelihood method (Figure 2). Based on the bootstrap values in the tree, the SAGs appeared to be divided into multiple distinct populations. The presence or absence of specific ARGs, plasmids, and virulence factors revealed different gene content profiles for each SAG, although they were all classified as the same species. Among the plasmids, p0111 was detected only in GHA9_AS_00007, which is a replicon type of the P1-like phage plasmid carrying the ESBL *bla*_CTX-M-55_ gene (Wang et al., 2022). This plasmid was also detected in GHA9_AS_00029, GHA9_AS_000173, and GHA9_AS_000205, which indicated that it was not a contaminant. An IncFIC-type plasmid was detected exclusively in GHA9_AS_00236, whereas IncFII-type plasmids were identified in all other SAGs. Moreover, many SAGs carried *bla*_CTX-M-15_, a globally recognized AMR marker and one of the most prevalent ESBL variants worldwide (Bevan et al., 2017); notably, *bla*_CTX-M-15_ in GHA9_AS_01225 was inserted within the chromosomally conserved *pgaABCD* locus (Itoh et al., 2008). To further validate the single-cell genomic findings, we additionally performed whole-genome sequencing on five *E. coli* isolates obtained from the same samples selected with A/S using culture-based methods. All five isolates carried *bla*_CTX-M-15_, consistent with the frequent detection of this gene in our single-cell genomic data. These results support the robustness of our approach in identifying *bla*_CTX-M-15_–harboring *E. coli* within the analyzed samples. Moreover, *anr*, *csgA*, *etsC*, *fimH*, and *gad*, which are used as indicators of diarrheagenic *E. coli* strains, were detected without gene-specific PCR testing.

### 3.3 Pan-genome analysis of representative SAGs

**Figure 3:**
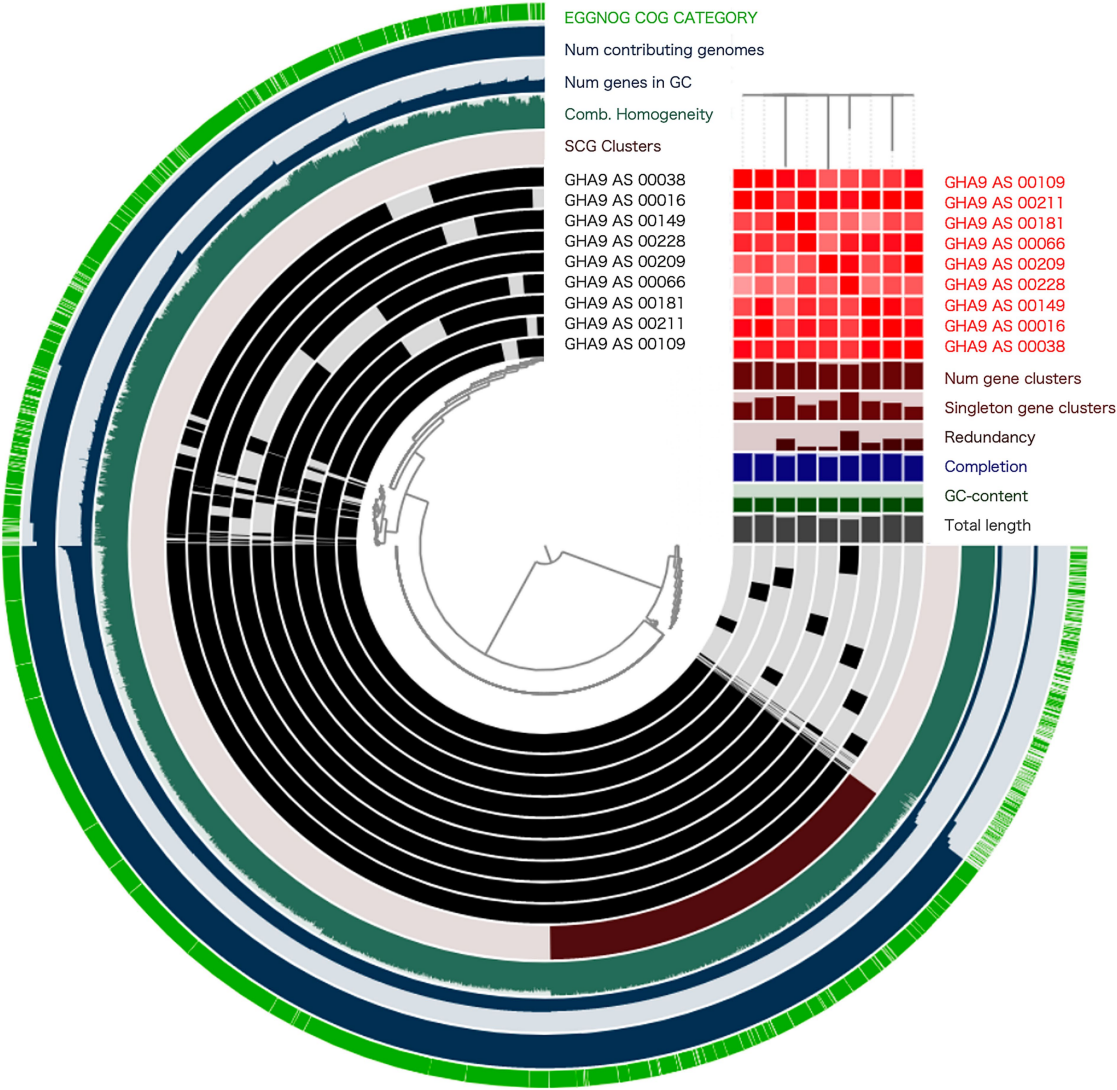
Pangenome analysis of nine representative SAGs. The analysis was performed using Anvi’o, based on genome sequences and gene prediction results obtained through eggNOG-mapper. The circular diagram integrates multiple layers of genomic information, including EGGNOG COG categories, gene cluster composition, homogeneity indices, and single-copy core gene (SCG) clusters. The inner phylogenetic tree reflects the presence or absence of each gastric cancer, and the outer one reflects the evolutionary rates. The central phylogenetic tree was constructed by calculating Euclidean distances between genomes based on gene presence/absence profiles and clustering them using Ward’s method, which corresponds to the default settings in Anvi’o. The ANI heatmap is scaled from 99% (white) to 100% (red).

Next, we selected nine representative SAGs (GHA9_AS_00209, GHA9_AS_00038, GHA9_AS_00181, GHA9_AS_00066, GHA9_AS_00109, GHA9_AS_00016, GHA9_AS_00149, GHA9_AS_00228, and GHA9_AS_00211) in which *bla*_CTX-M-15_ was detected with high completeness and conducted a pan-genome analysis based on gene clusters (GCs) to investigate genomic diversity. Among the nine SAGs, 4,814 GCs were identified, and 2,550 core GCs and 2,264 accessory GCs, including 605 single-copy GCs, were predicted, which indicated considerable variety not only in AMR and virulence factors but also in whole genomic profiles (Figure 3). Notably, the presence/absence circular diagram indicated functional variability among SAGs, suggesting specific adaptations and differences in gene content. These findings underscore the complexity of the *E. coli* pan-genome and provide insights into both the conserved and variable genomic elements.

### 3.4 Comparative functional analysis using COG classification

**Figure 4:**
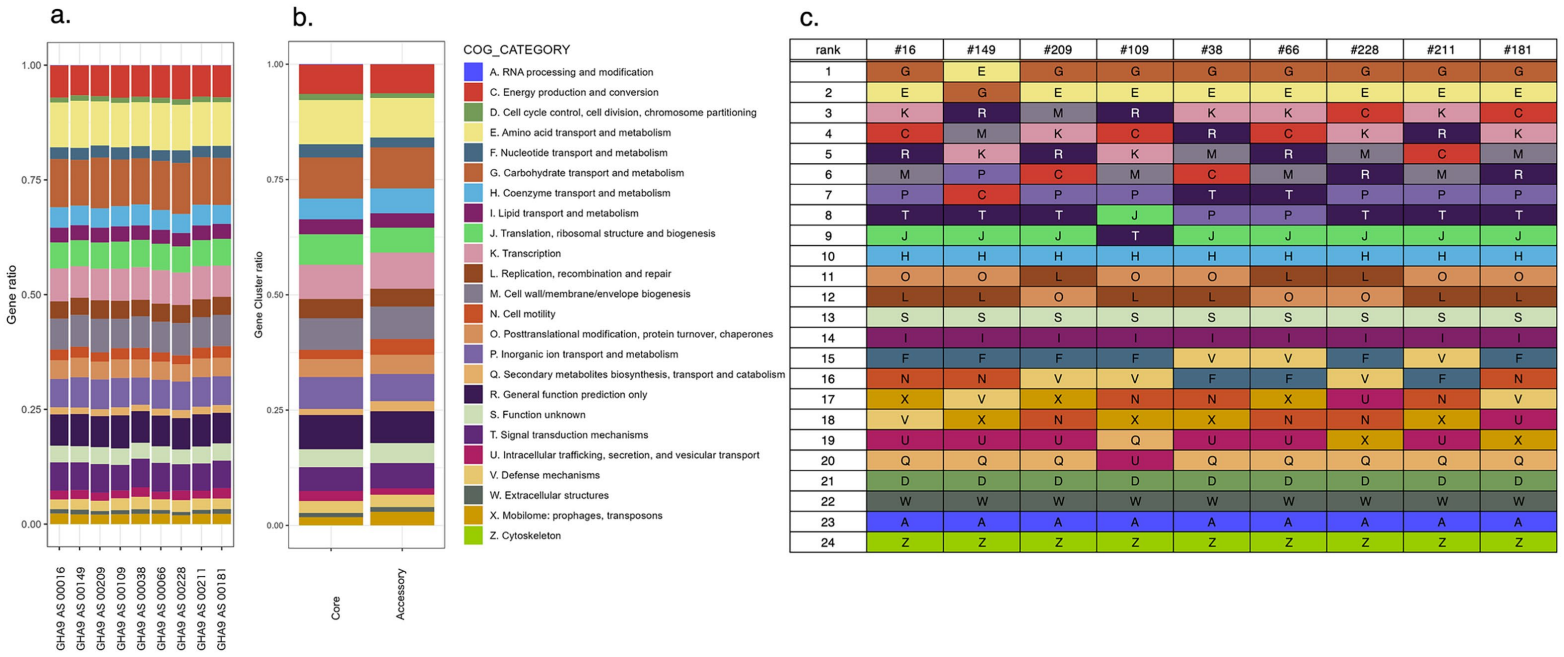
Cluster of orthologous genes (COGs) category profiles. **(a)** Ratio of each COG category on nine single-cell amplified genomes (SAGs). **(b)** Ratio of each COG category on core/accessory genes. **(c)** Descending order of the numbers of each COG categories.

Functional annotation based on COG classification, obtained by identifying sets of orthologous proteins across multiple complete genomes of bacteria and archaea, and allowing for the inference of conserved functions and evolutionary relationships, revealed distinct patterns of gene distribution across the analyzed samples (Figure 4a). The proportion of genes assigned to each COG category varied among the SAGs, indicating functional diversity. A comparative analysis of the COG category distributions between the core and accessory genes revealed a notable enrichment of specific functional categories within the accessory genome (Figure 4b). In particular, genes associated with mobilome—prophages and transposons (X)—and cell motility (N) increased in relative abundance by 1.23 and 1.48%, respectively, suggesting their potential roles in genomic plasticity, environmental adaptation, and niche-specific functions. Furthermore, we analyzed the distribution of COG categories by ranking them in descending order of the number of genes assigned to each category, allowing for the identification of the most functionally dominant groups across the dataset (Figure 4c). Categories related to metabolic processes, such as carbohydrate transport and metabolism (G) and amino acid transport and metabolism (E), were consistently represented across all samples. In contrast, categories associated with energy production and conversion (C) and cell wall/membrane/envelope biogenesis (M) were variably represented, indicating specific adaptations.

**Figure 5:**
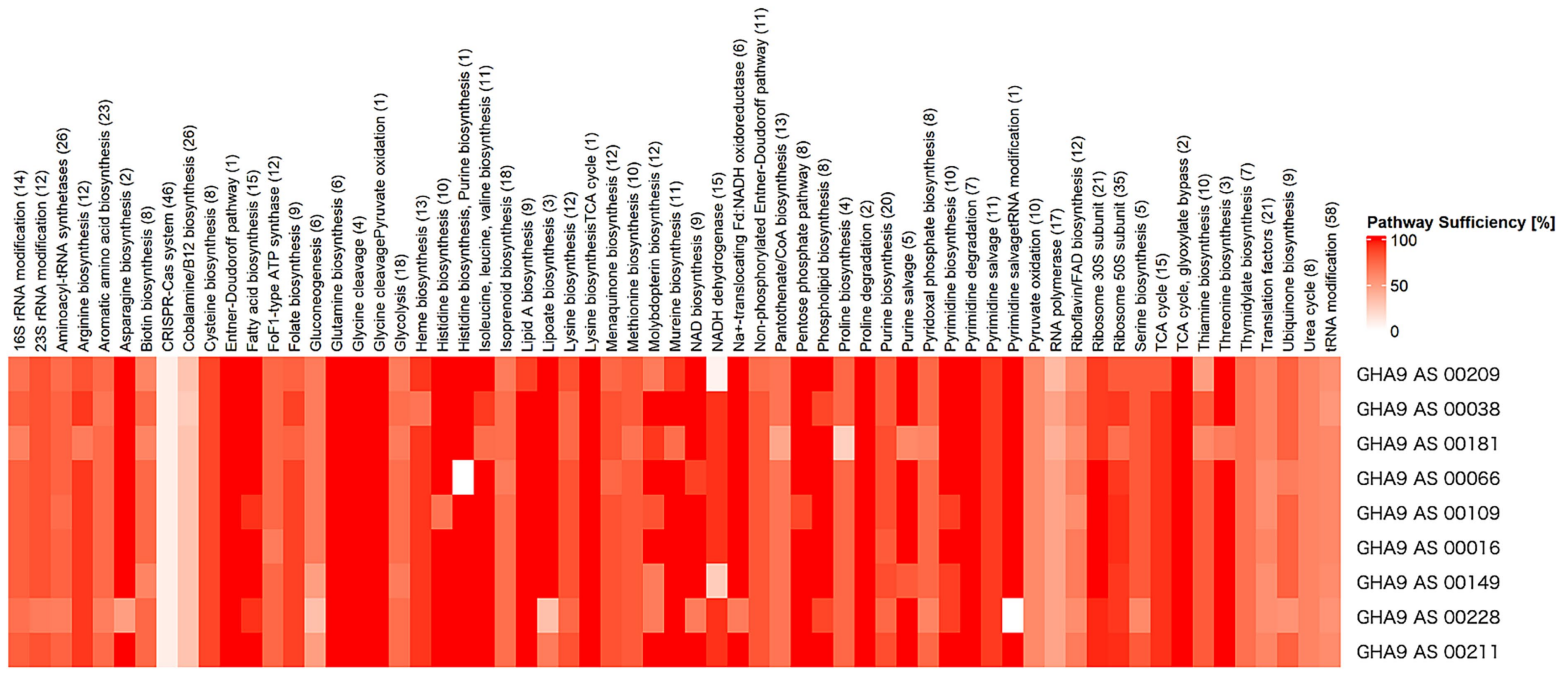
Cluster of orthologous genes (COGs) pathway sufficiency on nine single-cell amplified genomes (SAGs). The heatmap illustrates the percentage of sufficiency for each COG pathway, with darker red shades indicating higher levels of completeness. The numbers in parentheses indicate the number of genes assigned to each respective pathway.

We also analyzed the COG pathways of *E. coli* to determine the functional composition and potential metabolic capabilities of the genomes (Figure 5). Pathways, such as glycine cleavage and fatty acid biosynthesis, consistently exhibited high sufficiency across most samples, indicating that these core metabolic functions are well conserved. In contrast, pathways, such as nicotinamide adenine dinucleotide dehydrogenase (6.67–93.3%) and molybdopterin biosynthesis (66.7–100%) showed lower and more variable sufficiency levels, indicating potentially specific functional limitations or adaptations. These results highlight the functional heterogeneity among genomes and suggest that while essential metabolic pathways are largely intact, accessory functions related to biosynthesis and horizontal gene transfer may vary significantly between SAGs.

## 4 Discussion

This study highlights the critical utility of single-cell genomics in environmental AMR surveillance, particularly in complex and under-monitored ecosystems such as river water in Ghana. Unlike conventional metagenomic approaches, which are often not appropriate for associating ARGs with specific microbial taxa or genomic contexts, single-cell genomics enables direct linkage of AMR traits to individual microbial genomes. This precision is especially valuable in aquatic environments where microbial communities are highly diverse, and many species remain unculturable (Takhampunya et al., 2023). Our findings reveal that even within a single species, *E. coli*, substantial genomic and functional heterogeneity exists, including the presence of globally significant resistance genes, such as *bla*_CTX-M-15_, ESBL variants associated with clinical treatment failure. This indicates that even within *E. coli* strains possessing the same CTX-type genes, genetic diversity exists, which is challenging to detect using conventional culture methods or metagenomic approaches, highlighting the utility of single-cell genomic analysis.

The importance of AMR surveillance in Ghana is underscored by the high AMR-related mortality rate—27.3 deaths per 100,000 people (Bevan et al., 2017)—in this region and the documented presence of shared resistance genes across human, animal, and environmental sources (Fakorede et al., 2023). These findings align with the principles of the One Health framework, which emphasizes the interconnectedness of human, animal, and environmental health in addressing AMR threats (Jarocki et al., 2024). Genomic surveillance, particularly when integrated with advanced computational tools, has emerged as a cornerstone of One Health strategies, enabling the detection of resistance trends, outbreak predictions, and informed mitigation efforts. Recent studies have revealed the widespread circulation of ARGs, such as *bla*_CTX-M-15_, *bla*_NDM-1_, *bla*_OXA-48_, and *sul1*, across human, animal, and environmental sources in Ghana, underscoring the interconnected nature of AMR reservoirs in the region (Donkor et al., 2025). AMR bacteria have been detected in diverse human clinical specimens. The frequently reported species include *E. coli*, *Staphylococcus aureus*, *Klebsiella pneumoniae*, *Pseudomonas aeruginosa*, and *Streptococcus pneumoniae*. In animals, resistant strains have been isolated from various species with common pathogens, such as *Campylobacter* spp., *Arcobacter butzleri*, *Salmonella* spp., *Staphylococcus sciuri*, and *E. coli*. Environmental sources, such as hospital wastewater, rivers, and food contact surfaces, also harbor ARGs. Compared with countries with more advanced wastewater treatment infrastructure, such as Japan and parts of Europe, Ghana exhibits a higher prevalence and diversity of ARGs in environmental samples (Calland et al., 2023). A previous study on clinical isolates of multidrug-resistant *E. coli* obtained from rectal and hand swabs in a hospital located in one of the major cities in Ghana reported that *bla*_CTX-M-15_ was the most frequently detected ESBL gene and IncFIB was the most prevalent plasmid replicon type (Asare Yeboah et al., 2024), which is consistent with the findings of the present study.

The detection pattern of *qnrS1* mirrored that of *bla*_CTX-M-15_, which is consistent with previous reports, indicating that these ARGs were cotransferred via IncFIB-type plasmids (Thirumoorthy et al., 2023). The presence of IncFIB-type plasmids in nearly all the investigated SAGs further supports this hypothesis. Although *bla*_CTX-M-15_ and *qnrS1* were detected in both GHA9_AS_00123 and GHA9_AS_00228, IncFIB-type plasmids were not identified in these SAGs. However, annotation analysis of the contig containing *bla*_CTX-M-15_ and *qnrS1* in GHA9_AS_00123 revealed that these genes were inserted within the chromosomally conserved *pgaABCD* locus, suggesting that they are chromosomally located. Although *bla*_CTX-M-15_ is typically located on plasmids, whole-genome sequencing using conventional culture methods revealed that it can also be integrated into the chromosome (Shawa et al., 2021). The single-cell genomic approach employed in this study enables the simultaneous detection of genes located on both plasmids and chromosomes, representing a significant methodological advancement. Furthermore, despite the close genetic relationship between the IncFIC and IncFII replicon types, to the extent that IncFIC can substitute for IncFII in the RST formula of the pMLST scheme (Ruzickova et al., 2025), our single-cell analysis demonstrated a resolution sufficient to distinguish between these two replicons. This highlights the high precision of the method in detecting subtle differences in the composition of plasmid replicon.

*E. coli* has emerged as a significant carrier of AMR factors not only in clinical situations (Pitout and Laupland, 2008) but also in aquatic environments (Calarco et al., 2024). In this study, we detected diarrheagenic *E. coli* marker genes without performing gene-specific PCR. Furthermore, naturalized *E. coli* strains, which have adapted to survive and proliferate in engineered environments such as wastewater treatment plants, represent a significant and persistent reservoir of ARGs (Yu et al., 2022). Their ability to withstand conventional disinfection processes, including chlorination, UV irradiation, and heat, indicates that they may be co-evolving resistance mechanisms not only to antibiotics but also to water treatment interventions (Yu et al., 2022). In many conventional AMR surveys, only a single strain per species has been analyzed, resulting in an insufficient evaluation of intraspecies genetic diversity and functional variation. Moreover, *rpoS*, which is considered to contribute to stress responses under environmental conditions in many bacterial species (Bouillet et al., 2024), was consistently detected in all nine SAGs. In contrast, several genes were identified in only a subset of SAGs. This difference may reflect specific adaptations to distinct environmental niches. Multicopper oxidases are enzymes that possess multiple copper ions at their active sites and catalyze oxidation reactions. They have been implicated in various biological processes, including antibiotic biosynthesis, sporulation, copper tolerance, morphogenesis, and manganese oxidation (Rensing and Grass, 2003). The *cumA* gene encodes the multicopper oxidase, an aromatic hydrocarbon degradation pathway enzyme, which specifically catalyzes the first oxidation step in cumene catabolism (Francis and Tebo, 2001). Cumene tends to accumulate in environmental matrices, such as soil and groundwater, and *Pseudomonas* strains harboring *cumA* can utilize this compound as a carbon source. *CumA* is frequently located on plasmids, often in association with other catabolic genes; thus, it has the potential to be horizontally transferred to other Gram-negative bacteria via coresident or conjugative plasmids (Francis and Tebo, 2001). In this study, *cumA*, which belongs to the COG category “Q” that is represented among accessory genes, was detected in only one of the nine SAGs analyzed, which indicates that this *E. coli* strain may have acquired the capacity for cumene degradation via horizontal gene transfer. An analysis of the *cumA*-annotated sequence in GHA9_AS_00211 using the Basic Local Alignment Search Tool for Nucleotide revealed 100% identity with a genomic sequence from *Pseudomonas* species, suggesting possible horizontal transfer from *Pseudomonas* species. This exemplifies how the present approach can resolve fine-scale differences among strains within the same species. In the COG pathway analysis, the molybdopterin biosynthesis pathway sufficiency varied among the SAGs. Molybdopterin forms the central organic scaffold of the molybdenum cofactor and is essential for the catalytic activity of molybdoenzymes (Neumann et al., 2011). In *E. coli*, molybdenum cofactor-dependent enzymes, such as nitrate reductase, dimethyl sulfoxide reductase, formaldehyde dehydrogenase, and trimethylamine N-oxide reductase, play critical roles in nitrogen metabolism, anaerobic respiration, and organic compound degradation. These metabolic processes are vital for bacterial survival under various environmental conditions (Neumann et al., 2011). The biosynthesis of molybdopterin and its subsequent modification into molybdopterin guanine or cytosine dinucleotide cofactors are tightly regulated and enzyme-specific. As demonstrated in a previous study, mutations in the conserved motifs of nucleotidyltransferases MobA and MocA significantly impair cofactor formation and insertion into target enzymes, resulting in reduced molybdenum content and loss of enzymatic activity (Neumann et al., 2011). Variations in the pathway efficiency may directly influence the survival of bacterial strains and their adaptability to different ecological niches.

The SAG-gel platform used in this study offers significant advantages over conventional single-cell genome sequencing methods. In fluorescence-activated cell sorting-based single-cell genomic analysis, individual cells are sorted into multiwell plates using a flow cytometer, followed by cell lysis using alkaline solutions or similar reagents (Lasken, 2007). In principle, this approach enables the recovery of complete genomic information, including sequences of 16S rRNA genes and plasmids. However, challenges remain, including the difficulty in isolating single cells and the limited range of applicable lysis methods (Rinke et al., 2013). Droplet-based single-cell analysis methods other than SAG-gel have been developed, which allow rapid detection of specific pathogens (Luo et al., 2017). Nevertheless, owing to their immiscibility with the oil phase, these systems often face difficulties in performing multistep reactions, efficiently lysing both Gram-positive and Gram-negative bacteria, and removing WGA inhibitors (Hatzenpichler et al., 2020). Single-cell sorting and sequencing using Raman spectroscopy are also available. However, laser-based sorting may damage target bacteria, potentially resulting in a highly limited genome assembly (Xu et al., 2020). By encapsulating individual bacterial cells in agarose gel beads, SAG-gel enables multistep enzymatic lysis and WGA within a stabilized matrix, thereby reducing amplification bias and improving genome coverage by up to 25% compared with that achieved using alkaline lysis protocols (Nishikawa et al., 2022). This approach also facilitates the recovery of high-quality genomes from both Gram-positive and Gram-negative bacteria, including uncultured taxa from complex environmental samples, such as soil and seawater. Furthermore, fluorescence-based sorting of gel beads containing amplified DNA, rather than intact cells, enhances sorting efficiency and minimizes contamination, even in particle-rich samples (Hosokawa et al., 2022). Despite these strengths, SAG-gel is relatively labor-intensive and requires specialized microfluidic equipment and reagents, which may limit its scalability in resource-constrained settings. Additionally, although this method excels at strain-level resolution and functional annotation, the throughput and cost per genome are higher than those for bulk metagenomic approaches. It is important to recognize that the SAG-gel workflow does not yet accommodate all microbial types. Certain organisms, such as filamentous actinomycetes or fungi, cannot be effectively encapsulated because their cell dimensions or morphologies are incompatible with gel-bead formation. Moreover, genomes from high-GC microorganisms are often recovered at lower completeness due to amplification bias inherent to MDA-based WGA. Genome recovery is also reduced for taxa that are resistant to enzymatic lysis, as cells embedded in gel beads cannot undergo mechanical disruption (Chijiiwa et al., 2020; Nishikawa et al., 2022). Nevertheless, the precision and depth of genomic insight provided by SAG-gel justify its application in high-priority contexts such as environmental AMR surveillance, where accurate linkage of resistance genes to specific microbial hosts is essential. In the results of 16S rRNA gene amplicon sequencing, both genus *Escherichia-Shigella* and genus *Acinetobacter* were detected. However, in single-cell genome sequencing, majority of the SAGs were identified as genus *Escherichia-Shigella*, whereas genus *Acinetobacter* was not detected. This discrepancy may be attributed to methodological differences between the two approaches. The 16S amplicon sequencing involves the extraction of bacterial DNA from an entire sample without distinguishing between live and dead cells, thereby allowing the detection of DNA from nonviable or inactive bacteria. In contrast, the SAG-gel method employed in this study includes steps to remove extracellular DNA by washing.

The study has a few limitations. The number of samples was relatively small and restricted to a single time point, which may limit the generalizability of our findings. In addition, we focused on environmental samples, which prevented us from assessing potential relationships with clinical or animal sources. Although we identified several virulence-associated genes, this finding should not be interpreted as conclusive evidence of pathogenicity. Our analysis does not assess pathogenicity at the phenotypic level, and the presence of these genes indicates potential rather than confirmed virulence. The Densu River flows through the Greater Accra Region, one of the most densely populated and economically significant areas in Ghana. The detection of clinically important AMR bacteria in river water samples highlights the potential for the environmental dissemination of AMR through water systems. Notably, *E. coli* strains exhibiting resistance patterns similar to those found in human and animal isolates have been detected in drinking water sources in this region (Ahmed et al., 2022). This observation raises concerns about the potential for AMR transmission through water used for domestic and agricultural purposes, further emphasizing the need for integrated surveillance and intervention strategies under the One Health framework. Therefore, further data accumulation is necessary to improve the robustness of AMR detection systems. In the pan-genome analysis, SAGs of the same species with high completeness and low contamination were selected for use. This approach is expected to provide better insights into interspecies diversity and evolutionary dynamics. However, because these are not complete genomes, it is possible to overestimate singleton and accessory genes, as well as to underestimate core gene clusters.

In summary, the integration of single-cell genomics into environmental AMR surveillance may provide enhanced resolution and accuracy, particularly in resource-limited settings. To the best of our knowledge, this is the first study to investigate AMR in aquatic environments in Ghana using the SAG-gel method. The results potentially support the development of robust genomic databases and enhance our understanding of microbial ecology.

## Data Availability

The datasets analyzed in this study can be found in online repositories at the National Center for Biotechnology Information (NCBI) BioProject database under the accession number PRJNA473419.
