# Supplementary material for: Single-cell genomic profiling of antimicrobial resistance in Escherichia coli from the Densu River, Ghana
Source: Front Microbiol. 2026 Apr 23;17:1797725. doi: 10.3389/fmicb.2026.1797725 (PMC13149393; doi:10.3389/fmicb.2026.1797725)
Supplement: Supplementary file 1 [file Table_1.DOCX]

1. **Supplementary Table 1.** Data summary of 16S rRNA amplicon sequencing. Taxa that could not be classified at the genus level were labeled as Unassigned, and those with a relative abundance of less than 1% were grouped under Others.
