# Supplementary material for: Single-cell genomic profiling of antimicrobial resistance in Escherichia coli from the Densu River, Ghana
Source: Front Microbiol. 2026 Apr 23;17:1797725. doi: 10.3389/fmicb.2026.1797725 (PMC13149393; doi:10.3389/fmicb.2026.1797725)
Supplement: Supplementary file 2 [file Table_2.DOCX]

1. **Supplementary Table 2.** Statistics of single-cell metagenomic sequencing.
